# Supplementary material for: Multiple introductions of multidrug-resistant typhoid associated with acute infection and asymptomatic carriage, Kenya
Source: eLife. 2021 Sep 13;10:e67852. doi: 10.7554/eLife.67852 (PMC8494480; doi:10.7554/eLife.67852)
Supplement: Supplementary file 6. [file elife-67852-supp6.docx]

**Supplementary Table 6. Culture positive typhoid cases and asymptomatic carriers**

| **Typhoid Cases** | **Total** | **<=1 year** | **1-7 years** | **7-16 years** | **Linear regression association with age (p-value)** |
| --- | --- | --- | --- | --- | --- |
| Febrile participants subjected to  stool and blood culture | 4,670 | 630 | 3,299 | 741 | - |
| *S*. Typhi positive | 148 (3.2%) | 14 (2.2%) | 102 (3.1%) | 32 (4.3%) | 0.000525* |
| - Female | 49 (1.0%) | 6 (0.95%) | 36 (1.1%) | 7 (0.94%) | 0.536 |
| - Male | 99 (2.1%) | 8 (1.3%) | 66 (2.0%) | 25 (3.4%) | 0.0000773* |
| WGS confirmed *S*. Typhi positive | 100 (2.1%) | 7 (1.1%) | 67 (2.0%) | 26 (3.5%) | 0.0001* |
| - Female | 36 (0.77%) | 4 (0.63%) | 25 (0.76%) | 7 (0.94%) | 0.508 |
| - Male | 64 (1.4%) | 3 (0.48%) | 42 (1.27%) | 19 (2.6%) | 0.000009* |
| **Asymptomatic Carriers** | **8,549** | **641** | **5,495** | **2,413** | **-** |
| Total stool cultures from non-febrile  age-matched controls | 8,530 | 641 | 5,480 | 2,409 | - |
| *S*. Typhi positive | 95 (1.1%) | 4 (0.62%) | 62 (1.1%) | 29 (1.2%) | 0.402 |
| - Female | 46 (0.54%) | 3 (0.47%) | 31 (0.57%) | 12 (0.50%) | 0.585 |
| - Male | 49 (0.57%) | 1 (0.16%) | 31 (0.57%) | 17 (0.71%) | 0.081 |
| WGS confirmed *S*. Typhi positive | 55 (0.64%) | 4 (0.62%) | 31 (0.57%) | 20 (0.83%) | 0.262 |
| - Female | 29 (0.34%) | 3 (0.47%) | 16 (0.29%) | 10 (0.42%) | 0.909 |
| - Male | 26 (0.30%) | 1 (0.16%) | 15 (0.27%) | 10 (0.42%) | 0.127 |
