# Supplementary material for: Multiple introductions of multidrug-resistant typhoid associated with acute infection and asymptomatic carriage, Kenya
Source: eLife. 2021 Sep 13;10:e67852. doi: 10.7554/eLife.67852 (PMC8494480; doi:10.7554/eLife.67852)
Supplement: Supplementary file 7. [file elife-67852-supp7.docx]

**Supplementary Table 7 – Comparison of phenotypic and genotypic AMR profiles of 136 (n=128 H58, n=8 Non-H58) high quality *S.* Typhi genome sequences**

| **Drug_class** | **Resistant phenotype*** | **Resistant genotype** | **Very major error** | **Susceptible phenotype** | **Susceptible genotype** | **Major error** | **Sensitivity** | **Specificity** | **PPV** | **NPV** |
| --- | --- | --- | --- | --- | --- | --- | --- | --- | --- | --- |
| **Beta-lactamases** | | | | | | | | |  |  |
| Ampicillin | 100 | 95 | 5 (3.7%) | 36 | 28 | 8 (5.9%) | 95% | 77.8 % | 92.2% | 84.8% |
| Ceftazidime | 1 | 0 | 1 (0.74%) | 135 | 135 | 0 | 0% | 100% | 0% | 99.3% |
| Cefotaxime | 4 | 0 | 4 (2.9%) | 132 | 132 | 0 | 0% | 100% | 0% | 97.1% |
| Ceftriaxone | 1 | 0 | 1 (0.74%) | 135 | 135 | 0 | 0% | 100% | 0% | 99.3% |
| Cefpodoxime | 4 | 0 | 4 (2.9%) | 132 | 132 | 0 | 0% | 100% | 0% | 97.1% |
| **Chloramphenicol** | | | | | | | | |  |  |
| Chloramphenicol | 93 | 92 | 1 (0.74%) | 43 | 32 | 10 (7.4%) | 98.9% | 74.4% | 89.3% | 97.0% |
| **Tetracyclines** | | | | | | | | |  |  |
| Tetracycline | 69 | 65 | 4 (2.9%) | 67 | 59 | 8 (5.9%) | 94.2% | 88.1% | 89.0% | 93.7% |
| **Folate pathway inhibitors** | | | | | | | | |  |  |
| Co-trimoxazole | 98 | 96 | 2 (1.5%) | 38 | 31 | 7 (5.1%) | 98.0% | 81.6% | 93.2% | 93.9% |
| **Quinolones and Fluoroquinolones** | | | | | | | | |  |  |
| Ciprofloxacin | 63 | 55 | 8 (5.9%) | 73 | 41 | 32 (23.5%) | 87.3% | 56.2% | 63.2% | 63.2% |
| Nalidixic acid | 65 | 21 | 44 (32.4%) | 71 | 71 | 0 | 32.3% | 100% | 100% | 61.7% |

*indicates where resistant and intermediate phenotypes have been combined as resistant
