# Supplementary material for: Multiple introductions of multidrug-resistant typhoid associated with acute infection and asymptomatic carriage, Kenya
Source: eLife. 2021 Sep 13;10:e67852. doi: 10.7554/eLife.67852 (PMC8494480; doi:10.7554/eLife.67852)
Supplement: Supplementary file 8. [file elife-67852-supp8.docx]

**Supplementary Table 8 – Distribution of n=153 *S.* Typhi genotypes among each sex for cases and controls**

|  | **Female** | **Male** |
| --- | --- | --- |
| **WGS-confirmed cases** | **35** | **64** |
| EA1 | 12 (34.3%) | 23 (35.9%) |
| EA2 | 16 (45.7%) | 30 (46.9%) |
| EA3 | 4 (11.4%) | 11 (17.9%) |
| non-H58 | 3 (8.57%) | 0 |
| **WGS-confirmed carriers** | **28** | **26** |
| EA1 | 10 (35.7%) | 10 (38.5%) |
| EA2 | 18 (64.3%) | 9 (34.6%) |
| EA3 | 0 | 2 (7.69%) |
| non-H58 | 0 | 5 (19.2%) |
