# Supplementary material for: Multiple introductions of multidrug-resistant typhoid associated with acute infection and asymptomatic carriage, Kenya
Source: eLife. 2021 Sep 13;10:e67852. doi: 10.7554/eLife.67852 (PMC8494480; doi:10.7554/eLife.67852)
Supplement: Supplementary file 9. [file elife-67852-supp9.docx]

**Supplementary table 9 - Climatic predictors of elevated case and carrier counts for all samples**

| **Typhoid Cases** | | | | | | |
| --- | --- | --- | --- | --- | --- | --- |
| **Month** | **Same month** | | **Previous month** | | **2 months prior** | |
|  | **OR (95% CI)** | **p-value** | **OR (95% CI)** | **p-value** | **OR (95% CI)** | **p-value** |
| **Rainfall (precipitation)**  **> 75 mm** | 0.65 (0.13-3.2) | 0.72 | 0.85 (0.17-4.4) | 1 | 2.4 (0.46-17.1) | 0.29 |
| **Minimum temperature**  **>14°C** | 0.17 (0.025-0.86) | 0.022* | 0.39 (0.080-1.7) | 0.20 | 0.60 (0.13-2.6) | 0.53 |
| **Maximum temperature**  **>26°C** | 0.43 (0.094-1.8) | 0.22 | 0.43 (0.094-1.8) | 0.22 | 0.55 (0.12-2.3) | 0.52 |
| **Asymptomatic Carriers** | | | | | | |
| **Month** | **Same month** | | **Previous month** | | **2 months prior** | |
|  | **OR (95% CI)** | **p-value** | **OR (95% CI)** | **p-value** | **OR (95% CI)** | **p-value** |
| **Rainfall (precipitation)**  **> 75 mm** | 0.95 (0.19-4.5) | 1 | 1.18 (0.23-5.9) | 1 | 1.18 (0.23-5.9) | 1 |
| **Minimum temperature**  **>14°C** | 0.16 (0.029-0.74) | 0.0095* | 0.72 (0.17-3.1) | 0.75 | 1.1 (0.26-4.7) | 1 |
| **Maximum temperature**  **>26°C** | 0.19 (0.037-0.86) | 0.024* | 0.30 (0.063-1.3) | 0.11 | 0.82 (0.19-3.4) | 1 |

Values in cells are odds ratios and p-values for Fisher’s exact test between high case/control count and high rainfall/temperature. * highlights p-value <0.05.
