# Supplementary material for: Multiple introductions of multidrug-resistant typhoid associated with acute infection and asymptomatic carriage, Kenya
Source: eLife. 2021 Sep 13;10:e67852. doi: 10.7554/eLife.67852 (PMC8494480; doi:10.7554/eLife.67852)
Supplement: Supplementary file 10. [file elife-67852-supp10.docx]

**Supplementary table 10 - Climatic predictors of WGS confirmed elevated case and carrier counts inside the DSS**

| **Typhoid Cases** | | | | | | |
| --- | --- | --- | --- | --- | --- | --- |
| **Month** | **Same month** | | **Previous month** | | **2 months prior** | |
|  | **OR (95% CI)** | **p-value** | **OR (95% CI)** | **p-value** | **OR (95% CI)** | **p-value** |
| **Rainfall (precipitation)**  **> 75 mm** | 0.92 (0.076-6.91) | 1 | 4.71 (0.64-40.3) | 0.08 | 10.3 (1.3-133.7) | 0.011* |
| **Minimum temperature**  **>14°C** | 0.44 (0.055-3.1) | 0.41 | 0.98 (0.14-7.81) | 1 | 5.4 (0.56-275.2) | 0.21 |
| **Maximum temperature**  **>26°C** | 1.25 (0.18-9.89) | 1 | 0.30 (0.025-2.19) | 0.23 | 0.34 (0.029-2.47) | 0.41 |
| **Asymptomatic Carriers** | | | | | | |
| **Month** | **Same month** | | **Previous month** | | **2 months prior** | |
|  | **OR (95% CI)** | **p-value** | **OR (95% CI)** | **p-value** | **OR (95% CI)** | **p-value** |
| **Rainfall (precipitation)**  **> 75 mm** | 1.6 (0.12-16.8) | 0.63 | 0 (0-2.85) | 0.30 | 0 (0-2.85) | 0.30 |
| **Minimum temperature**  **>14°C** | 0.40 (0.030-4.01) | 0.37 | 0.45 (0.034-4.50) | 0.63 | 0.45 (0.034-4.50) | 0.64 |
| **Maximum temperature**  **>26°C** | 0.57 (0.043-5.63) | 0.65 | 0.57 (0.043-5.63) | 0.65 | 0.64 (0.048-6.29) | 1 |
