# Supplementary material for: Multiple introductions of multidrug-resistant typhoid associated with acute infection and asymptomatic carriage, Kenya
Source: eLife. 2021 Sep 13;10:e67852. doi: 10.7554/eLife.67852 (PMC8494480; doi:10.7554/eLife.67852)
Supplement: Supplementary file 11. [file elife-67852-supp11.docx]

**Supplementary table 11 - Climatic predictors of WGS confirmed elevated case and carrier counts for all samples collected**

| **Typhoid Cases** | | | | | | |
| --- | --- | --- | --- | --- | --- | --- |
| **Month** | **Same month** | | **Previous month** | | **2 months prior** | |
|  | **OR (95% CI)** | **p-value** | **OR (95% CI)** | **p-value** | **OR (95% CI)** | **p-value** |
| **Rainfall (precipitation)**  **> 75 mm** | 0.90 (0.16-4.50) | 1 | 1.08 (0.19-5.60) | 1 | 3.05 (0.59-16.9) | 0.15 |
| **Minimum temperature**  **>14°C** | 0.54 (0.12-2.43) | 0.50 | 0.41 (0.085-1.82) | 0.20 | 0.98 (0.22-4.52) | 1 |
| **Maximum temperature**  **>26°C** | 1.32 (0.30-6.10) | 0.75 | 0.86 (0.19-3.82) | 1 | 0.42 (0.084-1.86) | 0.32 |
| **Asymptomatic Carriers** | | | | | | |
| **Month** | **Same month** | | **Previous month** | | **2 months prior** | |
|  | **OR (95% CI)** | **p-value** | **OR (95% CI)** | **p-value** | **OR (95% CI)** | **p-value** |
| **Rainfall (precipitation)**  **> 75 mm** | 2.25 (0.35-13.7) | 0.41 | 0.27 (0.0054-2.52) | 0.40 | 0 (0-1.16) | 0.043* |
| **Minimum temperature**  **>14°C** | 0.79 (0.14-4.85) | 1 | 0.91 (0.16-5.51) | 1 | 0.51 (0.084-2.93) | 0.46 |
| **Maximum temperature**  **>26°C** | 0.67 (0.11-3.77) | 0.71 | 1.17 (0.21-7.1) | 1 | 1.32 (0.23-8.04) | 1 |
