# Supplementary material for: Multiple introductions of multidrug-resistant typhoid associated with acute infection and asymptomatic carriage, Kenya
Source: eLife. 2021 Sep 13;10:e67852. doi: 10.7554/eLife.67852 (PMC8494480; doi:10.7554/eLife.67852)
Supplement: Supplementary file 12. [file elife-67852-supp12.docx]

**Supplementary table 12 - Climatic predictors of elevated cases presenting at study clinics**

| **Presenting cases** | | | | | | |
| --- | --- | --- | --- | --- | --- | --- |
| **Month** | **Same month** | | **Previous month** | | **2 months prior** | |
|  | **OR (95% CI)** | **p-value** | **OR (95% CI)** | **p-value** | **OR (95% CI)** | **p-value** |
| **Rainfall (precipitation)**  **> 75 mm** | 0.90 (0.15-6.84) | 1 | 0.76 (0.12-5.89) | 1 | 0.64 (0.10-5.02) | 0.67 |
| **Minimum temperature**  **>14°C** | 0.16 (0.0032-1.47) | 0.11 | 0.19 (0.0028-1.28) | 0.061 | 0.62 (0.084-3.64) | 0.71 |
| **Maximum temperature**  **>26°C** | 0.48 (0.064-2.77) | 0.45 | 0.28 (0.024-1.79) | 0.15 | 0.28 (0.024-1.79) | 0.15 |
